# Autophagy Induced by Calcium Phosphate Precipitates Involves Endoplasmic Reticulum Membranes in Autophagosome Biogenesis

**DOI:** 10.1371/journal.pone.0052347

**Published:** 2012-12-21

**Authors:** Xi Chen, Min Li, Daohong Chen, Wentao Gao, Jun-Lin Guan, Massaki Komatsu, Xiao-Ming Yin

**Affiliations:** 1 Department of Pathology and Laboratory Medicine, Indiana University School of Medicine, Indianapolis, Indiana, United States of America; 2 Children’s Hospital, Zhejiang University School of Medicine, Hangzhou, Zhejiang, China; 3 Department of Surgery, University of Pittsburgh, School of Medicine, Pittsburgh, Pennsylvania, United States of America; 4 Department of Internal Medicine, University of Michigan Medical School, Ann Arbor, Michigan, United States of America; 5 Tokyo Metropolitan Institute of Medical Science, Tokyo, Japan; Cinvestav-IPN, Mexico

## Abstract

Calcium can play an important role in the regulation of autophagy. We previously reported that exogenously introduced calcium in the form of calcium phosphate precipitates (CPP) induces autophagy. Here we showed that CPP-induced autophagy required the classical autophagic machinery, including the autophagosome initiating molecules FIP200 and Beclin 1, as well as molecules involved in the autophagosome membrane extension, Atg4, Atg5 and Atg3. On the other hand, Atg9 seemed to place a restriction on CPP-induced autophagy. Loss of Atg9 led to enhanced LC3 punctation and enhanced p62 degradation. CPP-induced autophagy was independent of mTOR and reactive oxygen species. It also did not affect MAP kinase activation and ER stress. DFCP1 is an ER-resident molecule that binds to phosphatidylinositol 3-phosphate. CPP activated DFCP1 punctation in a class III phosphatidylinositol-3-kinase and calcium dependent manner, and caused the association of DFCP1 puncta with the autophagosomes. Consistently, ER membranes, but not Golgi or mitochondrial membranes, colocalized with CPP-induced LC3 positive autophagosomes. These data suggest that CPP-induced autophagosome formation involves the interaction with the ER membrane.

## Introduction

Macroautophagy (referred to as autophagy hereafter) is a self-digestion process with intrinsic functions in the maintenance of cellular homeostasis. Under physiological conditions, it constitutes a major part of the catabolic process, and helps to eliminate misfolded or unfolded proteins and damaged organelles [1]. By up-regulation of autophagy, cells can handle various hazardous conditions. The initiation and completion of autophagy is controlled by a series of autophagy-related (Atg) proteins, which is manifested in the biogenesis and maturation of the double-membraned autophagosome. In the yeast, there are 32 identified Atg proteins, many of which have homologues in mammalian cells [2], [3]. In yeast, a group of Atg proteins forms pre-autophagosomal structure (or phagophore assembly site, PAS) to initiate autophagy. In mammals, the early autophagosome is named as isolation membrane (IM), which seems to be derived from multiple membrane sources depending on the nature of the stimuli.

In mammalian cells, mTOR is a key upstream negative regulator of autophagy. Common autophagy stimuli, including starvation and rapamycin, activate autophagy through inhibition of mTOR. Two protein kinase complexes are typically involved in the initiation process: the Atg1/Unc-51-like kinase (ULK) 1/2 complex (UKC) and the Atg6/Beclin 1/class III phosphatidylinositol-3-kinase (PI-3K) complex. mTOR regulates the activity of UKC, which is also composed of Atg13, Atg101, and FIP200/Atg17, to affect the Beclin 1 complex. Beclin 1/Atg6 interacts with the Class III PI-3 kinase (consisting of Vps34 and Vps15) and Atg14/Barkor to promote the production of phosphatidylinositol 3-phosphate (PI3P). The autophagy effectors of PI3P can include WIPI-1/Atg18-Atg2 complex and DFCP1, which is an ER-Golgi residential protein. Consistently, Atg14 was found to be the DFCP1 recruiter in the ER [4], suggesting the contribution of ER membrane to the biogenesis of autophagosome. PI3P is also required for the elongation of the autophagosome, which depends on two ubiquitin (Ub)-like enzyme systems. One system is comprised of Atg12 (ubiquitin-like), Atg7 (E1-like) and Atg10 (E2-like), and promotes the conjugation of Atg12 to Atg5, which further binds to Atg16. The other system is comprised of microtubule-associated protein 1 light chain 3 (LC3)/Atg8 (ubiquitin-like), Atg7 (E1-like) and Atg3 (E2-like), and functions to conjugate LC3/Atg8 to phosphatidylethanolamine (PE). Lipidation of LC3/Atg8 is important for the maturation of PAS/IM into double-membraned autophagosomes. Atg4 is a cysteine protease that processes the LC3/Atg8 molecule to allow its conjugation with PE [5]. Finally, transmembrane protein Atg9 seems to shuttle between different membrane compartments and PAS/IM, and participates in autophagosome biogenesis as well as substrate degradation [6], [7], [8], [9].

The source of the contributing membrane to PAS/IM remains controversial. ER, Golgi [10], plasma membrane [11] and mitochondria inner membrane [12], [13] have all been proposed to contribute to autophagosome membranes [14]. The DFCP1 protein has both ER-residing and PI3P-binding domains, and is located in membrane compartment associated with autophagosome biogenesis [15], suggesting that ER membrane could contribute to early autophagosomal membranes. This notion is further substantiated by electron tomography studies in which an ER subdomain is found to connect to, and cradle the newly formed IM [16], [17].

Intracellular calcium is mainly stored in the ER lumen, and can be released upon stimulation to serve as a second messenger in cell growth and cell death. Calcium can regulate autophagy in both positive and negative ways [18], [19]. Small amounts of calcium spontaneously released from ER are picked up by the mitochondria to maintain bioenergetic production of ATP, which inhibits AMPK and therefore autophagy in normal growing cells [20]. Disturbance of this process by reducing intracellular calcium level could in turn trigger autophagy [19], [21]. Increased cytosolic calcium released from ER or extracellular space could also promote autophagy [19], [22]. We and others have found that exogenously introduced calcium in the form of calcium phosphate precipitate (CPP) can potently induce autophagy [23], [24], [25]. CPP is the first used chemical reagent to facilitate DNA transfection [26]. It forms complex with DNA and bring it into the cells. We had found that this process also triggers a transient autophagy induction, which is usually resolved in 24 hours [23]. However, DNA is not needed for CPP to induce autophagy and the ability relies on calcium as it can be inhibited by BAPTA-AM, an intracellular calcium inhibitor. CPP can induce a very unique LC3-positive tubular-vesicular structure, which may be related to its biogenesis and/or maturation [24]. However, the signaling pathway and the potential membrane source of the autophagosomes induced by CPP remain unknown. The aim of this study is thus to determine how CPP induces autophagy and autophagosome biogenesis.

## Results

### CPP-induced Autophagy is Dependent on the Core Autophagy Machinery

**Figure 1:**
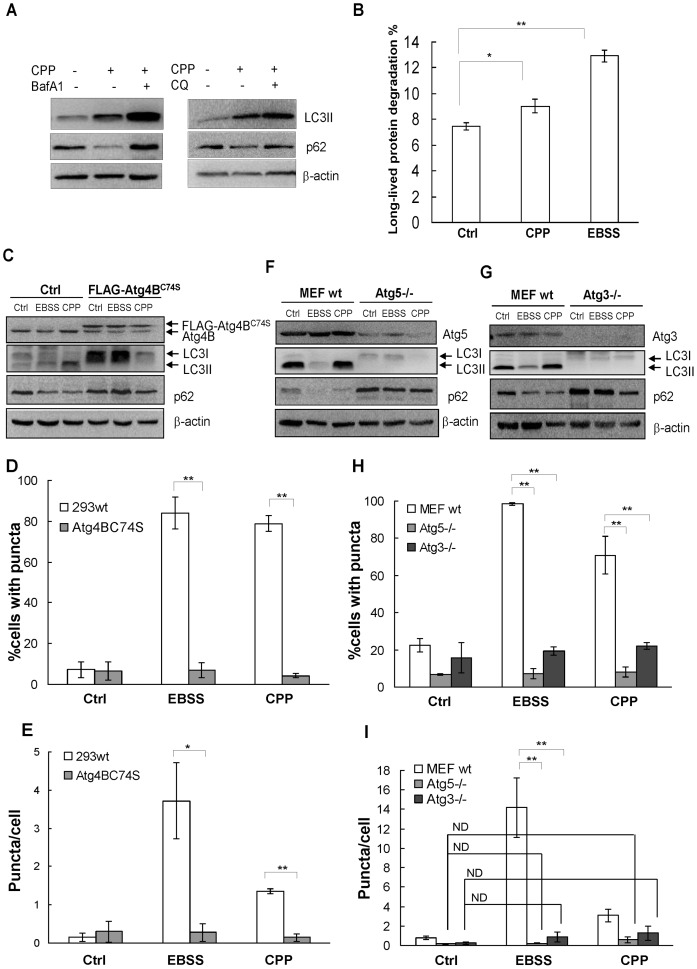
CPP-induced autophagy is dependent on the conjugation system. (**A–B**) Wild-type MEF were treated with CPP with or without CQ (50 µM) or BafA1 (500 nM), or were starved by incubating in EBSS as a positive control for autophagy initiation, followed by Western blotting for LC3 and p62 (A), and long-lived protein degradation assay (B). (**C–E**) HEK293 cells stably expressing FLAG-Atg4B^C74S^ or an empty vector were treated with EBSS or CPP and analyzed by Western blotting (C) and quantification of GFP-LC3 puncta (D, E). (**F–I**) Wild-type, Atg5−/−, and Atg3−/− MEFs were transduced with adenoviral GFP-LC3. Forty-eight hours later cells were treated with EBSS or CPP. Selected Atg proteins and p62 were detected by Western blotting (F, G). GFP-LC3 puncta were quantified (H, I). Western blots shown are from one representative experiment of two to three performed for each panel. Quantitative data (mean±SEM) are from 3–5 experiments. *: *P*<0.05, **: *P*<0.01.

We have shown that CPP activated autophagy in multiple cell lines [23], [24]. In MEF, autophagy induction could be clearly demonstrated by the increased amount of lipidated LC3, i.e., LC3II, and the decrease of p62, one of the selective substrates for autophagy (Figure 1A). Long-lived protein degradation increased from 7.45% in the control group to 9.03% in the CPP group. Bafilomycin A1 (BafA1) or chloroquine (CQ), both lysosome inhibitors, could block CPP-induced autophagy degradation as manifested by an even higher level of LC3II in the presence of these chemicals (Figure 1A). Consistently, the level of p62 returned to the control level. As a comparison, induction of autophagy by EBSS (starvation) also resulted in p62 reduction (Figure 1C) and increased long-lived protein degradation (Figure 1B). The LC3II level was not significantly detected in EBSS-treated cells (Figure 1C and below) due to increased autophagic degradation, which could be reversed by BafA1 (Figure S1) [27].

To examine whether the CPP effect was dependent on the core autophagy machinery, we studied several autophagy-deficient cell lines. First, a dominant negative mutant of Atg4B, Atg4B^C74S^, which caused hampered lipidation of LC3 and GFP-LC3 punctation [28] (Figure 1C), was introduced to the HEK293 cells. p62 degradation was blocked after both EBSS and CPP treatment in cells expressing Atg4B^C74S^ (Figure 1C). In cells stably expressing GFP-LC3, we measured both ‘the average number of puncta per cell’ and ‘the percentage of cells with puncta’. The former represents the scale of autophagic response, while the latter indicates the overall response of cells to autophagy stimulation. The number of GFP-LC3 puncta increased following either EBSS or CPP treatment in normal cells, but not in cells expressing Atg4B^C74S^ (Figure1D, E). Similarly, in Atg3−/− and Atg5−/− MEF cells, CPP also failed to increase LC3II (Figure 1F, G) and GFP-LC3 punctation (Figure 1H, I). The p62 levels were only slightly decreased in Atg3−/− and Atg5−/− MEFs after treatment of CPP, compared to that in the wild type cells (Figures 1F–G, Fig. S2).

**Figure 2:**
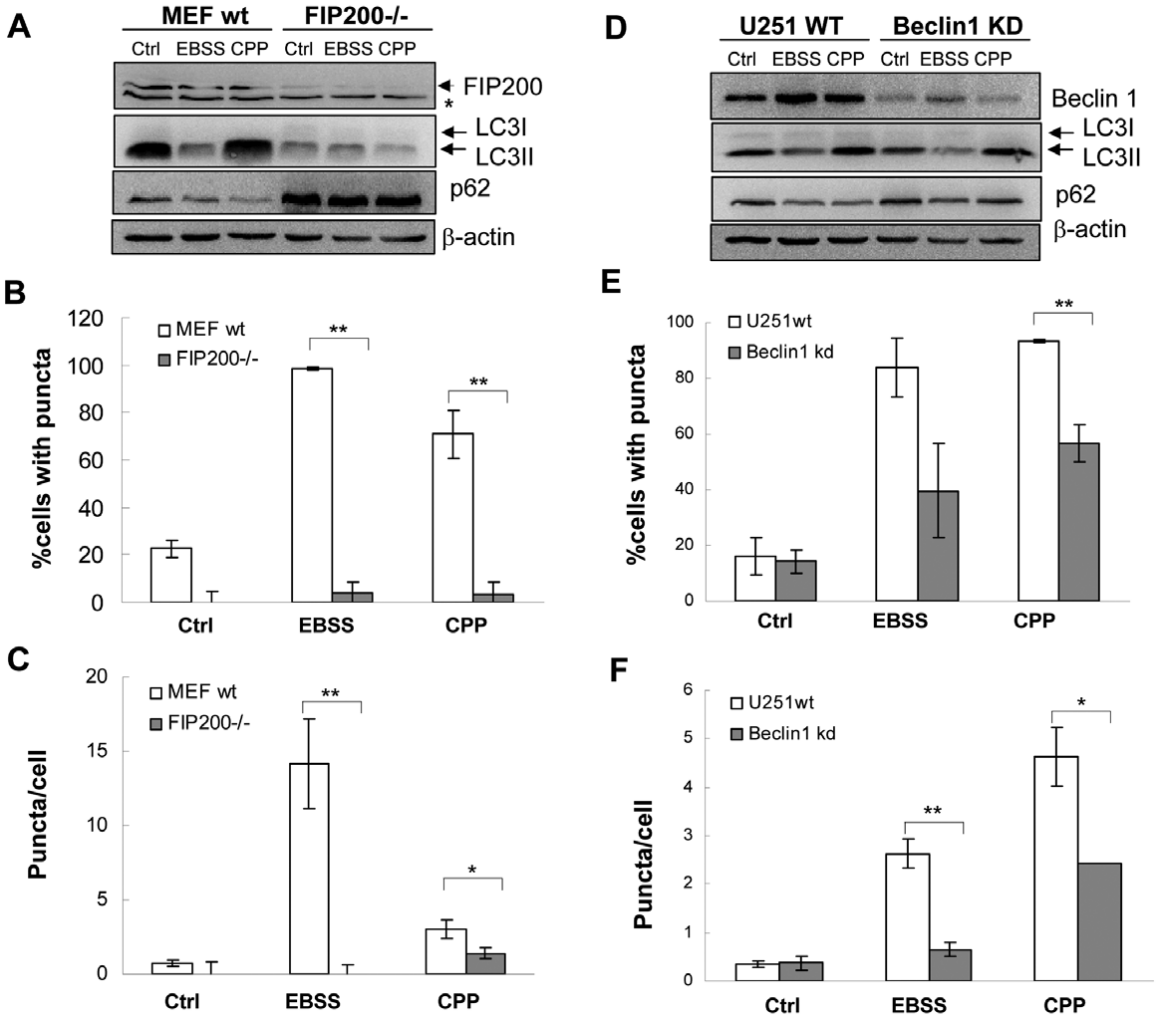
CPP-induced autophagy is dependent on FIP200 and Beclin 1. Wild-type and FIP200−/− MEFs (**A–C**), and Beclin 1 wild type and Beclin 1-low U251 cells (**D–F**) stably expressed GFP-LC3. They were treated with EBSS or CPP, followed by Western blotting analysis (A, D) and quantification of GFP-LC3 puncta (B, C, E, F). Asterisk in A indicates a non-specific band. Western blots shown are from one representative experiment of two to three performed for each panel. Quantitative data (mean±SEM) are from 3–5 experiments. *: *P*<0.05, **: *P*<0.01.

In starvation-induced-autophagy, the UKC (ULK1/FIP200/Atg13 complex) is thought to be involved at an early time point of autophagosome formation. This complex then activates the Beclin 1/Atg14/class III PI-3K complex. To determine whether CPP-induced autophagy relies on the same initiation molecules, we examined MEF deficient of FIP200 expression and a glioblastoma cell line U251, in which Beclin 1 was constitutively knocked-down [29]. In FIP200−/− cells stimulated with CPP the formation of LC3II was decreased compared to FIP200 wild type cells (Figure 2A). It was noted that p62 level was overall increased in FIP200−/− cells due to suppression of autophagy, but neither EBSS nor CPP induced a reduction of p62 in this cell line. Consistently EBSS or CPP did not induce GFP-LC3 punctation in FIP200-deficient cells (Figure 2B, C).

The endogenous LC3II level was high in the wild type U251 cells and in the subline with Beclin 1 knockdown (KD) (Figure 2D). However, quantification based on exogenously introduced GFP-LC3 allowed the discrimination between the wild type and KD cells in their response to EBSS and CPP (Figure 2E, F). The KD cells did not respond with as much GFP-LC3 punctation as the wild type cells. Similarly, the p62 level was reduced in WT cells but not in Beclin 1 KD cells following EBSS or CPP treatment (Fig. 2D). These results were consistent with our previous findings with the use of transient Beclin 1 knockdown or the PI3- kinase inhibitor, 3-MA, in HEK293 cells [23]. Taken together, these findings indicated that CPP-induced autophagy required the same set of initiating and conjugating Atg molecules as starvation-induced autophagy.

### Atg9 could Play a Significant Role in Limiting CPP-induced Autophagy

**Figure 3:**
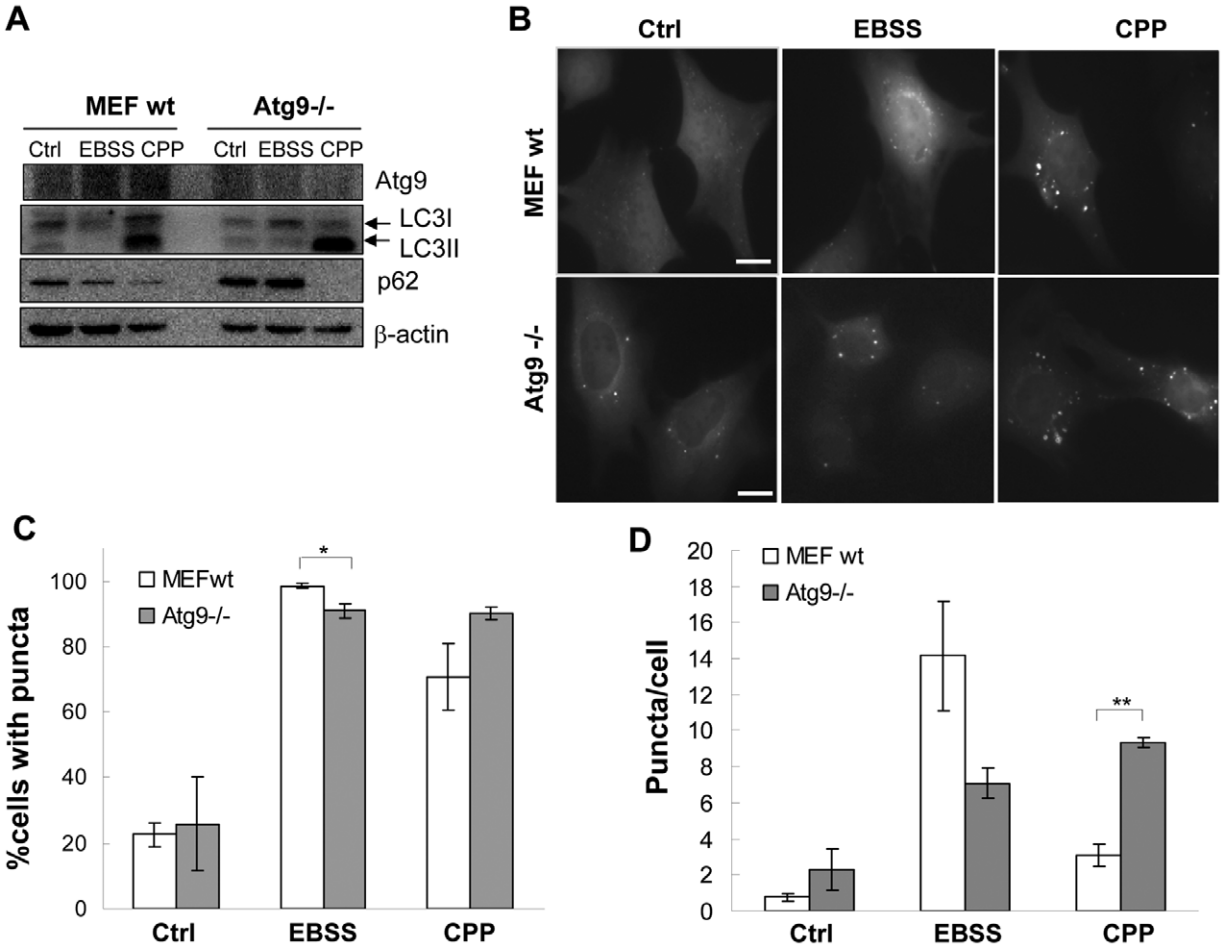
Atg9 is inhibitory for CPP-induced autophagy. Atg9 wild type and Atg9-deficient MEFs stably expressing GFP-LC3 were treated with EBSS or CPP followed by Western blotting analysis (**A**) and quantification of GFP-LC3 puncta (**B–D**). Scale bars: 10 µm in B. Western blots shown are from one representative experiment of two to three performed for each panel. Quantitative data (mean±SEM) are from 3–5 experiments. *: *P*<0.05, **: *P*<0.01.

Atg9 is a unique molecule in the autophagy machinery. As the only membrane spanning Atg molecule, it may transport membranes between different compartments to participate in autophagosome biogenesis [7]. We thus examined the dependency of CPP-induced autophagy on Atg9. Compared to Atg9 wild type MEF, Atg9-deficinet (Atg9−/−) MEF [30] has a lower LC3II and a higher p62 level at basal status (Figure 3A). EBSS promoted LC3II and p62 degradation in wild type MEF, but not in Atg9−/− MEF. In Atg9 wild type and deficient cells stably expressing GFP-LC3 (Figure 3B–D), the background level of GFP-LC3 punctation was higher in Atg9−/− MEF. After EBSS treatment, although the percentage of cells with GFP-LC3 puncta was increased to the same level in both genotypes, the number of puncta per cell was fewer in Atg9−/− MEF. The data thus suggested an overall weakened autophagic response to EBSS in Atg9−/− cells, as reported previously [7], [30]. In contrast CPP caused a higher LC3II increase as well as more p62 reduction than EBSS did, especially in Atg9−/− MEF (Figure 3A). CPP also caused more GFP-LC3 punctation in Atg9−/− cells (Figure 3B–D). We also observed a larger size of GFP-LC3 puncta in Atg9−/− cells (Figure 3B). This was not an artifact of GFP tagging since we confirmed the observation by immuno-staining for the endogenous LC3 (Figure S3). These findings suggested that in the absence of Atg9, there was increased LC3 positive autophagosome biogenesis resulting in increased degradation.

### CPP-induced Autophagy is Independent of the mTOR Pathway

**Figure 4:**
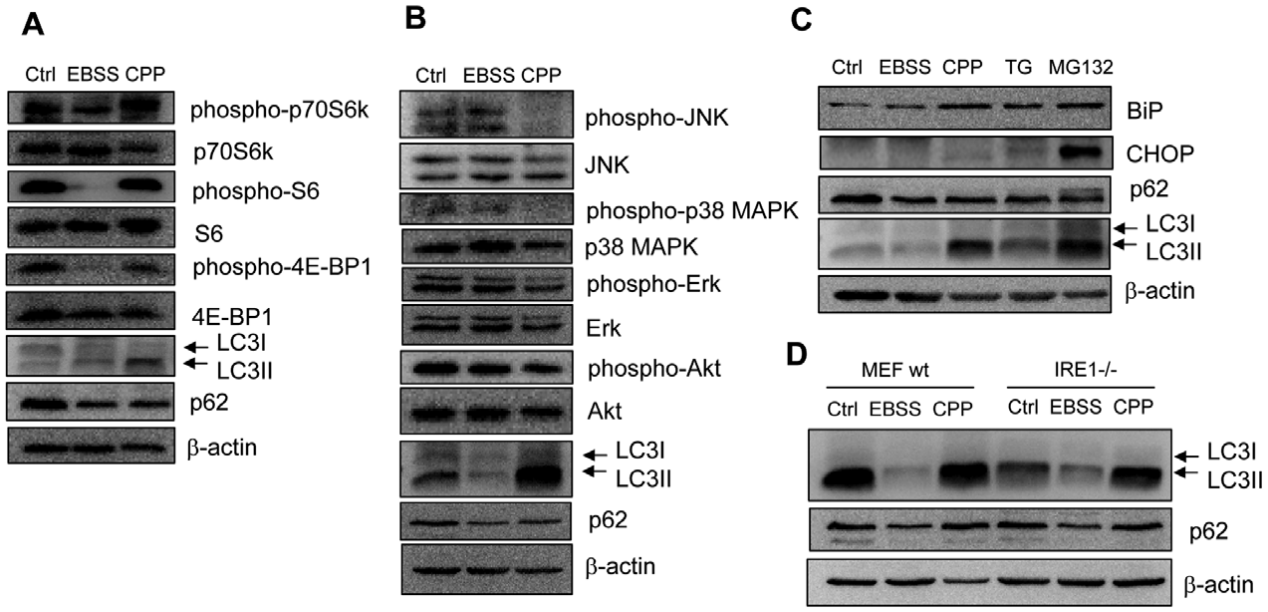
CPP-induced autophagy is independent of the signaling of mTOR, MAPK and ER stress. (**A–C**) HEK293 cells were treated with EBSS or CPP, thapsigargin (TG, 0.5 µM, 16h) or MG132 (1 µM, 16 h), followed by Western blotting with indicated antibodies. (**D**) Wild-type and IRE1−/− MEFs were treated with EBSS or CPP, followed by Western blotting with indicated antibodies. Western blots shown are from one representative experiment of two to three performed for each panel.

To determine signaling pathways activated by CPP, which lead to the engagement of the core autophagy machinery, we examined whether CPP inhibits the mTOR activity, commonly seen in autophagy activation [31], [32], [33]. In our experiments, starvation caused mTOR suppression as determined by the reduced phosphorylation of major mTOR downstream targets, *i.e*., p70S6 kinase, S6, and 4E-BP1 in 293 cells (Figure 4A) or in MEFs (Figure S4). However, there were no significant changes in the phosphorylated forms of these proteins following CPP treatment in both cell lines. These data indicated that CPP-induced autophagy could be independent of the mTOR pathway.

### CPP does not have Significant Impact on Mitogen-activated Protein Kinase (MAPK), ER Stress and Reactive Oxygen Species (ROS) Production

MAPK activation, ER stress and ROS are known to mediate the initiation of autophagy [34], [35], [36], [37]. However, we found no significant changes in the phosphorylation patterns of JNK, p38 MAPK, Erk and Akt following CPP treatment in HEK293 cells (Figure 4B). Thapsigargin (TG) is an inhibitor of sarco/endoplasmic reticulum Ca^2+^-ATPase (SERCA), while MG132 inhibits proteasome activity; both can activate autophagy through the induction of ER stress [38]. BiP and CHOP are chaperon proteins with up-regulated expression during ER stress. In HEK293 cells, a significant increase of BiP and CHOP was observed following TG and MG132 administration, but not with EBSS (Figure 4C). Following CPP treatment, BiP was increased but the change of CHOP was trivial. We further tested the ability of CPP to induce autophagy in IRE1−/− MEFs, which had a poor unfolded protein response to ER stress stimulation and were resistant to ER stress-induced autophagy [39]. We found no difference in LC3 lipidation between IRE1 wild type and deficient MEFs following CPP treatment (Figure 4D), suggesting that CPP-induced autophagy is not mediated by ER stress.

Carbonyl cyanide m-chlorophenylhydrazone (CCCP), a mitochondrial proton gradient uncoupling agent, can induce autophagy in a ROS-dependent manner, as it could be blocked almost completely by an anti-oxidant, N-acetyl cysteine (NAC) [36], in MEF and in HEK293 cells (Figure S5). However, NAC did not modify GFP-LC3 punctation induced by CPP, suggesting that ROS over-production may not be involved in CPP-induced autophagy.

### CPP-induced Autophagosomes is Associated with ER, but not Golgi Apparatus or Mitochondria

In order to determine the contributing membrane of the autophagosome formed after CPP stimulation, we investigated the association of CPP-induced autophagosomes with several subcellular organelles that had been implicated in autophagosome biogenesis: ER, Golgi apparatus and mitochondria [10], [13], [15]. We examined markers of the three organelles, including GM130 for Golgi complex and MitoTracker for mitochondria. For ER, five markers were used, including Sec61b, calnexin, ER Tracker, a CFP-tagged ER residing peptide containing an ER insertion signal and a KDEL C-terminal ER retention signal (CFP-ER), and DFCP1, an ER-residing, PI3P binding molecule [40].

**Figure 5:**
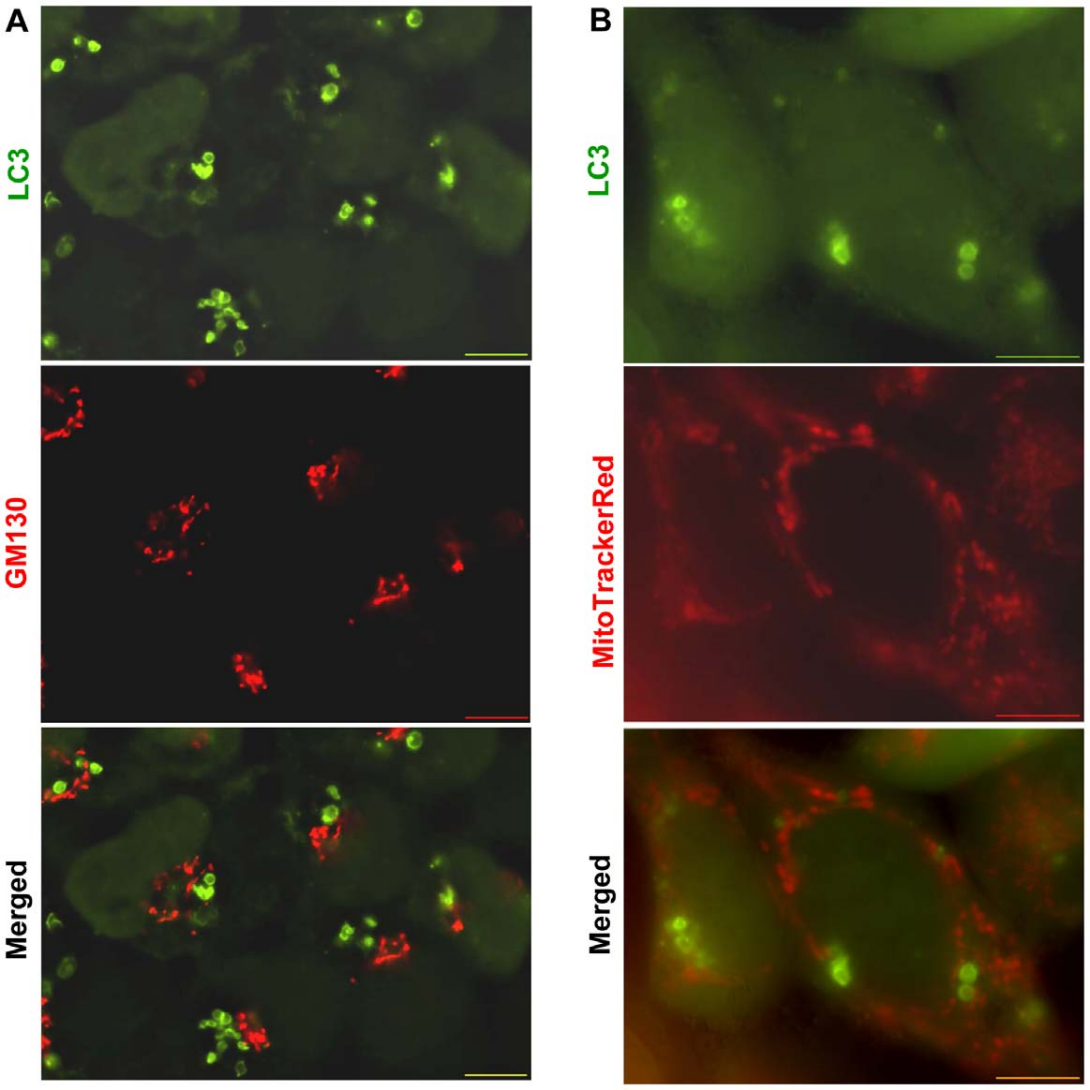
GFP-LC3-positive autophagosomes induced by CPP do not colocalize with the Golgi apparatus or the mitochondria. (**A**) HEK293 cells stably expressing GFP-LC3 were treated with CPP, fixed and stained with anti-GM130 and Cy3-conjugated secondary antibody. (**B**) The same cell line was labeled with MitoTracker Red (20 nM) 30 min prior to CPP treatment. Images of the CPP-treated cells were acquired through z-sectioning and deconvoluted. Scale bars: 10 µm.

Many of the CPP-induced GFP-LC3 puncta have a distinctive morphology with rings and tubules that we have named as LC3 positive tubulovesicular structures (LC3-TVS) [24]. We found that LC3-TVS did not colocalize with GM130 (Figure 5A). But the two markers were usually in close spatial vicinity. We also did not observe that CPP could induce Golgi fission, a phenomenon previously reported in autophagic cells under starvation [9]. On the other hand, CPP-induced LC3-TVS were not colocalized with the mitochondria, which were defined by the MitoTracker dye (Figure 5B).

**Figure 6:**
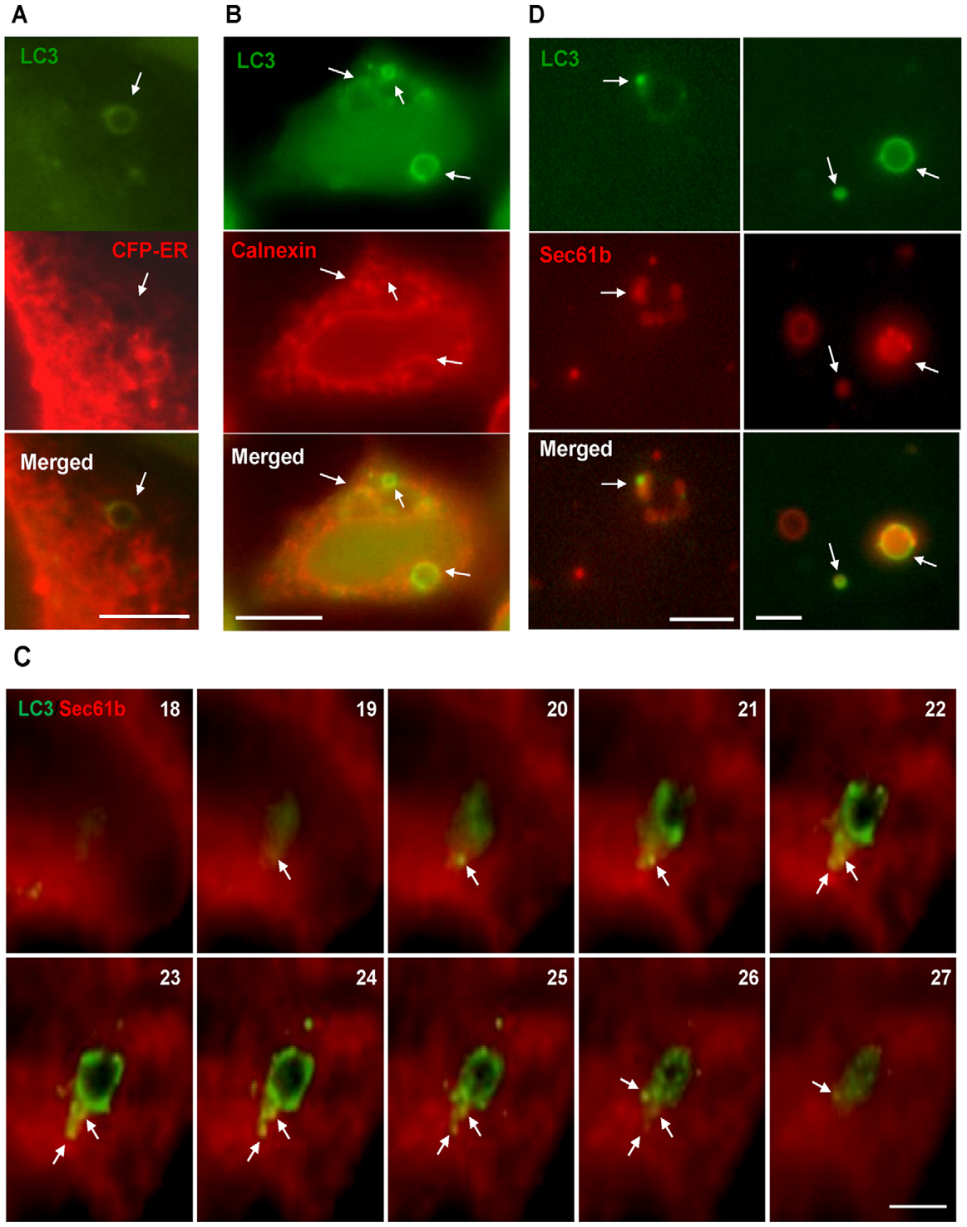
GFP-LC3 positive autophagosomes induced by CPP interact with ER membranes. (**A**) HEK293 cells stably expressing both GFP-LC3 and CFP-ER were treated with CPP. Fluorescent images were taken and CFP channel was pseudo-colored in red. (**B**) HEK293 cells stably expressing GFP-LC3 were treated with CPP, then fixed and stained with an anti-calnexin antibody and then Cy3-conjugated secondary antibody. (**C**) HEK293 cells stably expressing both GFP-LC3 and Sec61b-mCherry was treated with CPP, images were taken at different z-sections (the section is indicated) and deconvoluted. Arrows indicate the colocalization of LC3 and Sec61b signals. Constructed z-stacks are shown in movie S1. (**D**) A single clone of HEK293 cells stably expressing both GFP-LC3 and Sec61b-mCherry was treated with CPP and then lysed. Fluorescence images of the lysates were acquired. Vesicles showing the presence of both GFP-LC3 and Sec61b-mCherry were observed. Scale bars: 5 µm in A, C and D; 10 µm in B.

To determine the relationship of CPP-induced LC3-TVS with ER membrane, we used two different approaches to label the ER network to examine its relationship with CPP-induced GFP-LC3-TVS: calnexin, and CFP-ER. LC3-TVS were found to at least partially colocalize with each of the two markers (Figure 6A–B, Figure S6A–B). Similar observations were made with the use of the ER Tracker dye (data not shown).

We then examined cells with a double expression of GFP-LC3 and Sec61b-mCherry with the latter labeling the ER network. We found that LC3-TVS were tightly associated with Sec61b-positive membranes. Analysis of a series of images acquired through a range of z-sections showed that only a part of the GFP-LC3 TVS interacted with Sec61b-positive membranes (Figure 6C, Figure S6C, Movies S1–S2), with the rest of LC3 TVS being cradled along the Seb61b-positive membranes. These findings were consistent with electron tomographic studies indicating that LC3 membranes interact with ER network [16], [17].

To further document that LC3-TVS and ER membranes could be found in the same membrane compartment, we lysed cells co-expressing GFP-LC3 and Sec61b-mCherry following CPP treatment. Vesicles with both Sec61b and LC3 could be easily spotted in the lysate (Figure 6D). Quantification of the fluorescence-labeled vesicles revealed a significant increase of double positive vesicles after CPP treatment. In the control group, 5.7% of the vesicles were positive for both GFP-LC3 and Sec61b-mCherry, while in the CPP treated group, this number increased to 23.7%. Taken together, these observations indicated that CPP-induced autophagosome was closely related to ER membranes, but not with the Golgi apparatus or the mitochondria.

**Figure 7:**
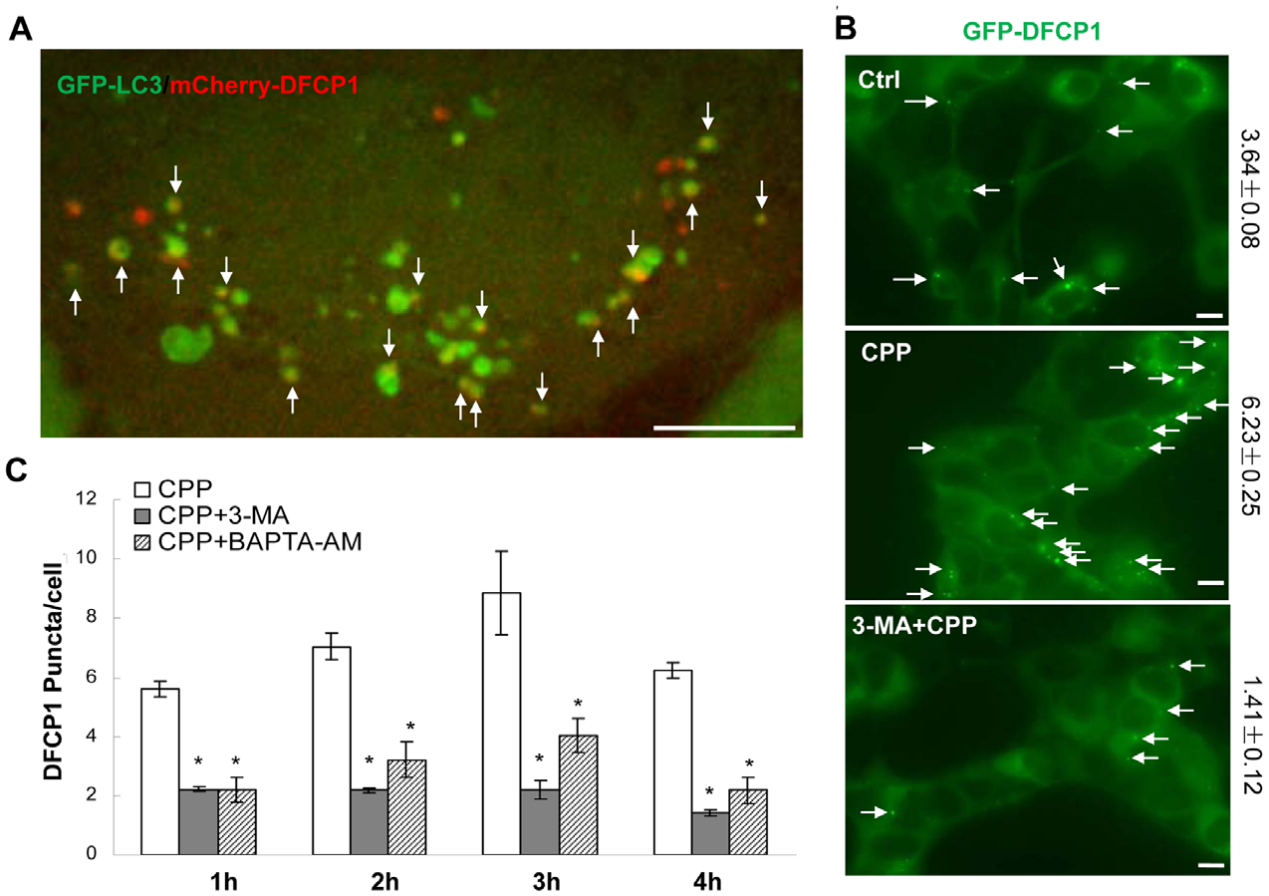
GFP-LC3 positive autophagosomes induced by CPP are associated with DFCP1 signals. (**A**) HEK293 cells stably expressing both GFP-LC3 and RFP-DFCP1 were treated with CPP and images of live cells were taken by confocal microscopy. (**B–D**) HEK293 cells stably expressing GFP-DFCP1 was treated with CPP with or without 3-MA (10 mM) (B–C), or BAPTA-AM (10 mM) (C) 30 min prior to CPP. GFP-LC3 puncta were quantified (B–C). Scale bars: 5 µm in A; 10 µm in B. Quantitative data (mean±SEM) are from 3–5 experiments. *: *P*<0.05, **: *P*<001.

Previous studies have shown that under starvation, DFCP1, an ER-residing PI3P binding molecule, could be recruited to a membrane compartment related to autophagosome biogenesis [4], [15], [41]. We found that DFCP1-positive puncta were increased by CPP treatment in a time-dependent manner (Figure 7A). The PI-3K inhibitor, 3-MA, significantly decreased DFCP1 punctation (Figure 7B, C), indicating that CPP-induced PI-3K activation resulted in an increased level of PI3P, which was required for DFCP1 recruitment. Consistently, the intracellular calcium chelator, BAPTA-AM, also significantly blocked DFCP1 punctation (Figure 7C). The CPP-induced DFCP1 puncta were associated with LC3-TVS (Figure 7A). DFCP1 puncta could be entirely wrapped inside LC3-rings or only partially overlapped with LC3 rings or tubules. These patterns could suggest a dynamic membrane interaction between the autophagosomes and the DFCP1 positive ER compartments depending on calcium and PI-3K activity.

## Discussion

### CPP Activates the Classical Autophagic Machinery by an mTOR Independent Pathway

To induce autophagy, exogenously introduced calcium needs to be in the form of calcium phosphate precipitate. Calcium chloride at the same concentration does not induce autophagy (data not shown). CPP needs to enter into cells for its effect to take place, which can be blocked by BAPTA-AM [23]. CPP enters cells likely through endocytosis [26]. However, its fate has not been clearly elucidated. When it forms complex with DNA as used for cellular transfection, the complex is presumably released into the cytosol where DNA could enter into the nucleus. It is possible that this context confers the condition under which CPP can activate autophagy, although the actual step leading to autophagy is yet to be determined.

Autophagy under nutrient is generally mediated by the mTOR pathway, which had been implicated in certain models of calcium-mediated autophagy regulation [19]. We thus investigated mTOR activities in CPP-induced autophagy in HEK293 and MEF cells. EBSS culture reduced the phosphorylation rates of two downstream target molecules of the mTOR signaling cascade, p70S6k and 4E-BP1, confirming mTOR inhibition upon starvation. CPP did not change the phosphorylation status of these proteins. In addition, there were also no changes in the phosphorylation of Akt, which can serve as an important positive upstream signal for mTOR. Therefore, mTOR is unlikely a target of CPP.

In HEK293 cells, we also examined several other MAP kinases including JNK (phosphorylated by SEK1 or MKK4), p38 MAPK (target of MKK3, MKK6, and SEK) and Erk (target of MEK) since they had been directly or indirectly implicated in several other studies regarding calcium-mediated autophagy regulation [19]. But we found that their phosphorylation status was not affected by CPP (Figure 4B). Finally, neither ER stress nor ROS seemed to be important in CPP-induced autophagy. The fact that CPP does not activate these signals fits to the fact that CPP is not a toxic reagent, and cells always recover from the initial stimulation of CPP-mediated transfection.

While the signaling mechanism has yet to be fully determined, we do find that the core autophagy machinery is engaged by CPP stimulation. The data suggest an essential role of UKC in CPP-induced autophagy as well as in starvation. Autophagy was suppressed in FIP200−/− MEFs (Figure 2A–C) and in ULK1−/− MEF cells (data not shown). We have also shown that CPP requires Beclin 1 using transient knockdown in HEK293 cells [23] and using constitutive knockdown in U251 cells (Figure 2D–F). Consistently, 3-MA, a PI-3K inhibitor, also suppressed CPP-induced autophagy [23]. Finally, this and our earlier study [23] together also indicate that CPP requires the LC3 conjugation molecules including Atg4, Atg5 and Atg3 to induce autophagy.

p62 is accumulated in autophagy deficiency cells at the basal level. We demonstrated that short-term treatment of CPP (4–5h) resulted in p62 degradation to the same degree as the starvation treatment, along with an increased LC3 lipidation, both of which could be reversed by CQ or BafA1 (Figure 1A). CPP also increased long-lived protein degradation (Figure 1B), although not as significant as in starvation. The difference in the magnitude of p62 degradation and long-lived protein degradation may suggest that the two parameters do not necessarily measure the autophagy capacity with the same sensitivity. The long-lived protein degradation assay could be more quantitative than the p62 western blot assay, particularly during autophagy induction [42], [43]. Taken together, these results indicated that CPP activates a productive autophagy process via the classical autophagy machinery.

### Atg9 may Prevent Excessive Autophagy under CPP Treatment

Atg9 in the yeast participates in the formation of PAS and is required for the onset of autophagy [44], [45]. The function and the mechanism of the mammalian Atg9 in autophagy have not been fully defined yet. It exists in several subcellular compartments and is thought to traffic between them, particularly between the Golgi complex and the endosomal membranes. The trafficking of Atg9 has been found to be controlled by molecules including p38 MAPK interacting protein [46], Bif1 [9], Atg13 [7], SNARE molecules [5], Atg1 and myosin [47]. Atg9 also interacts with Atg2 and Atg18. Formation of Atg9-positive tubular structures has been considered important in autophagosome biogenesis [5], [9]. In our previous studies, Atg9 was found to colocalize with LC3 after CPP treatment with an unevenly distributed pattern [24]. We speculated that Atg9 could be associated with specific docking sites on the autophagosomes and facilitate membrane exchange that regulates the maturation of autophagosomes.

In the present study, we found that consistent with previous work [30], starvation-induced autophagy still required the participation of Atg9. On the other hand, CPP elicited an increased autophagic activity in Atg9−/− MEF, as demonstrated by increased LC3 lipidation and dramatically decreased p62 level (Figure 3A), which could be reversed by CQ (data not shown). The greater reduction was not due to redistribution of p62 into an insoluble compartment in Atg9−/− cells (data not shown). These observations indicated that Atg9 may act as a negative regulator in CPP-induced autophagy to prevent an excessive autophagy. We speculate that Atg9, in addition to its known effects in promoting autophagy, may also prevent excessive autophagy by recycling off membrane components required for autophagosome biogenesis. This hypothesis of the dual effect of Atg9 would deserve a further investigation in future studies.

### Autophagosomes Stimulated by CPP Interact with the ER Membranes

Our study indicated that CPP does not induce over-production of ROS. The mitochondria remain structurally intact after CPP treatment (Figure 5B). Furthermore, CPP-induced LC3-TVS was not associated with the mitochondria. Thus CPP-induced autophagy would unlikely involve mitochondrial damage. The Golgi apparatus is considered to contribute to autophagosome biogenesis in yeast [10]. In our experiments, the Golgi marker was not associated with CPP-induced LC3-TVS (Figure 5A). In addition, unlike starvation, CPP did not induce Golgi fission. Taken together, it seems that the Golgi complex and the mitochondria may not be the major contributing membrane source in the CPP model.

Although ER stress does not seem to contribute to CPP-induced autophagy, ER membrane seems to be closely related to autophagosomes induced by CPP. We have used multiple approaches to document this close relationship, which identify the ER network by different mechanisms. Our observations are consistent with previous observations of starvation-induced autophagy in which isolation membranes can be cradled by ER membranes [16], [17]. Furthermore, ER-residing molecule DFCP1 has been shown to participate in the starvation-induced autophagy [15]. Our result showed that DFCP1 is a downstream effector of CPP, and the signaling requires the activity of PI-3K and depends on free calcium. DFCP1-positive ER membranes could contribute to LC3-TVS in a controlled and regulated manner. In starvation-induced autophagy, Atg14 was shown to recruit PI-3K to ER and induced DFCP1 punctation during autophagy [4]. ER-residing Bcl-2 was also proposed to regulate autophagy via interaction with Beclin 1 [22], [48]. Whether Atg14 and Bcl-2 are direct mediators in CPP-induced autophagy needs to be explored, but the importance of the Beclin 1/Atg14/PI-3K complex is clearly demonstrated in this model as discussed above.

In summary, we found that short-term treatment of CPP induces autophagy in an mTOR independent manner, and requires classical autophagic machinery including autophagosome nucleation molecules Beclin 1 and FIP200, as well as the conjugation molecules Atg4, Atg5 and Atg3. CPP does not produce ER stress or mitochondrial damage. ER membrane components, but not the mitochondria or the Golgi apparatus, could contribute to autophagosomes/LC3-TVS induced by CPP. CPP could serve as a valuable model agent to study autophagy initiation and autophagosome biogenesis due to its unique interactions with the cellular membranes.

## Materials and Methods

### Regents and Antibodies

All chemicals used were supplied by Sigma, Fisher and RPI. The following antibodies were used: GFP (Santa Cruz), β-actin (Sigma), calnexin (Santa Cruz), GM130 (BD Transduction), LC3 (MBL),Atg5 (Cell Signaling), Atg4B (Abgent), Atg3 (Abgent), Atg9 (Abcam), Beclin 1 (Santa Cruz), FIP200 (GeneTex), p62/SQSTM1 (MBL), total and phosphorylated (T421/S424) P70S6 kinase (Cell Signaling), total and phosphorylated (Ser235/236) S6 (Cell Signaling), total and phosphorylated (Thr37/46) 4E-BP1 (Cell Signaling), total and phosphorylated (Thr183/Tyr185) JNK (Cell Signaling), total and phosphorylated (Thr180/Tyr182) p38 MAPK (Cell Signaling), total and phosphorylated (Thr202/Tyr204) Erk (Cell Signaling), total and phosphorylated (Ser473) Akt (Cell Signaling), GADD153/CHOP (Santa Cruz), BiP (Sigma). Secondary antibodies were conjugated to HRP or Cy3 (Jackson ImmunoResearch). The rabbit polyclonal anti-LC3-II antibody was made using a peptide representing the NH2-terminal 14 amino acids of human LC3-II and an additional cysteine (PSEKTFKQRRTFEQC) [38].

### Cell Culture and Imaging

Atg9−/−MEF and U251 cells with constitutive knockdown of Beclin 1 were constructed as previously described [29], [30]. HEK293, MEF and U251 cells were maintained in DMEM medium supplemented with 10% fetal bovine serum, at 37°C with 5% CO_2_. CPP was prepared by adding CaCl_2_ drop wise to Na_2_HPO_4_ (in Hepes, pH 7.05) as previously described [23]. CPP 20% (v/v) was administrated to the cells 4 hours before analysis. To induce autophagy by starvation, cells were incubated in EBSS for 4 hours. MitoTracker (50 nM) and ER-Tracker (500 nM) (Molecular Probes, Invitrogen) were added to medium 30 min before subsequent treatment.

Fluorescence microscopy was carried out with a Nikon Eclipse TE200 epifluorescence microscope equipped with the NIS-Elements software. For colocalization studies, Z-stacks were obtained and digitally deconvoluted. Alternatively, confocal microscopy was carried out with a Perkin-Elmer R2–E2 equipped with Andor iQ software. GFP-LC3 puncta and puncta-containing cells are visually identified from the fluorescence images. For manual quantification of the puncta, at least 3 optical fields with over 50–200 cells per experimental condition were analyzed. Data from repeated experiments are subjected to statistical analysis.

### Immunoblotting and Immunostaining

Cells were washed with PBS and lysed in RIPA buffer supplemented with standard protease inhibitors as well as phosphatase inhibitors (1 mM Na_3_VO_4_, 1 mM β-glycerophosphate and 1 mM sodium pyrophosphate). Twenty (20) µg of protein per sample was subjected to SDS-PAGE and transferred to PVDF membranes. The membranes were blotted with the indicated antibodies and developed with SuperSignal West Pico chemiluminescent substrates (Pierce, Rockford, IL). The images were acquired by Kodak Image Station 4000 MM with the companion software (Carestream Health, Inc) with an exposure duration from 30 seconds to 10 minutes depending on signal strength. Digital images may be subjected to minor adjustment for brightness and contrast as a whole.

Cells were cultured on glass slides or in glass bottom culture dishes (MatTek Corporation), washed in PBS and fixed with 4% paraformaldehyde for 15 min at room temperature. Cells were then permeabilized, blocked with 1% BSA and incubated with primary and secondary antibodies. Cells were co-stained with Hoechst 33342 for the nuclei.

### Long-lived Protein Degradation

The assay was carried out as described previously [49]. Briefly, MEF were cultured in DMEM in 24-well plate, L-[^14^C]-valine was added to a final concentration of 0.2 µCi/ml to label the intracellular proteins. Cells were incubated for 18 hours at 37°C before changing to fresh medium for another hour at 37°C to degrade short-lived proteins. Long-lived protein degradation was then determined following treatment with CPP or EBSS for another 4 hours.

### Statistical Analysis

Data shown are the mean ± SEM from multiple experiments as indicated in figure legends. Data were subjected to t-test or one-way ANOVA using SigmaStat 3.5 (SYSTAT). *P*<0.05 was considered significant.

## Supporting Information

Figure S1 **Flux analysis EBSS-induced autophagy in MEF.** Wild-type MEF was cultured in EBSS for 1 h, 2 h and 4 h, with or without BafA1 (1 µM), followed by Western blotting for indicated proteins. Note the increase accumulation of LC33 in the presence of BafA1, which blocked the lysosomal degradation of the autophagosomes. This data showed the reduced level of LC3II in starved cells at later time points was due to increased autophagic degradation.
(TIF)

Figure S2 **Quantitative measurements of LC3II and p62 in normal and autophagy deficient cells. (A–B)** 293 cells expressing FLAG-Atg4B^C74S^ or vector (control) were treated with CPP or EBSS. The levels of LC3II (A) or p62 (B) were determined by immunoblotting shown in Figure 1C, and were standardized to that of β-actin. **(C–D)** MEFs of different genotypes were treated with CPP or EBSS. The level of LC3II (C) or p62 (D) was determined by immunoblotting shown in Figure 1F and 1G, and were standardized to that of β-actin. Quantitative densitometry analysis was carried out from 2–4 independent experiments. Data were presented as mean±SEM. *: p<0.01.
(TIF)

Figure S3 **Atg9 is inhibitory for CPP-induced autophagy.** Atg9 wt and Atg9−/− MEFs were treated with EBSS or CPP and then fixed. Formation of the endogenous LC3 puncta was detected by anti-LC3 immunostaining, counterstained with Hoechst 33342 for the nuclei. Scale bars: 10 µm.
(TIF)

Figure S4 **CPP-induced autophagy is independent of the signaling of mTOR.** MEFs were treated with EBSS or CPP. Cells were solubilized on site in the presence of protease inhibitor and phosphatase inhibitors, followed by Western blotting with indicated antibodies.
(TIF)

Figure S5 **CPP-induced autophagy is independent of the signaling of ROS.** **(A)** HEK293 cells stably expressing GFP-LC3 were treated with CPP or CCCP (40 µM) with or without NAC (10 mM) for 4 h. Images were acquired by fluorescence microscopy. Scale bars: 50 μm. (**B**) GFP-LC3 puncta was quantified from one representative experiment of three performed (mean ±SD). The data indicated that CCCP-induced, but not CPP-induced GFP-LC3 puncta formation could be blocked by NAC. Scale bars: 50 μm. *: p<0.01, ND: no statistical differences.
(TIF)

Figure S6 **GFP-LC3 positive autophagosomes induced by CPP interact with ER membranes.** **(A)** HEK293 cells stably expressing both GFP-LC3 and CFP-ER were treated with CPP. Fluorescent images were taken and CFP channel was pseudo-colored in red. **(B)** HEK293 cells stably expressing GFP-LC3 were treated with CPP, then fixed and stained with an anti-calnexin antibody and then Cy3-conjugated secondary antibody. **(C)** HEK293 cells stably expressing both GFP-LC3 and Sec61b-mCherry was treated with CPP. Images were taken at different z-sections (the section is indicated) and deconvoluted. Constructed z-stacks are shown in movie S2. Scale bars: 10 μm in A–B; 3 μm in C. Arrows indicate the colocalization of LC3 with CFP-ER, calnexin or Sec61b signals.
(TIF)

Movie S1 **HEK293 cells stably expressing both GFP-LC3 and Sec61b-mCherry was treated with CPP.** Images were taken at different z-sections, deconvoluted and converted to the movie. Z-section depth: 0.5 μm per frame. Scale bar: 10 µm in movie 1 and 6 µm in movie 2.
(AVI)

Movie S2 **HEK293 cells stably expressing both GFP-LC3 and Sec61b-mCherry was treated with CPP.** Images were taken at different z-sections, deconvoluted and converted to the movie. Z-section depth: 0.5 μm per frame. Scale bar: 10 µm in movie 1 and 6 µm in movie 2.
(AVI)
