# Supplementary figures and images for: Autophagy Induced by Calcium Phosphate Precipitates Involves Endoplasmic Reticulum Membranes in Autophagosome Biogenesis
Source: PLoS One. 2012 Dec 21;7(12):e52347. doi: 10.1371/journal.pone.0052347 (PMC3528773; doi:10.1371/journal.pone.0052347)

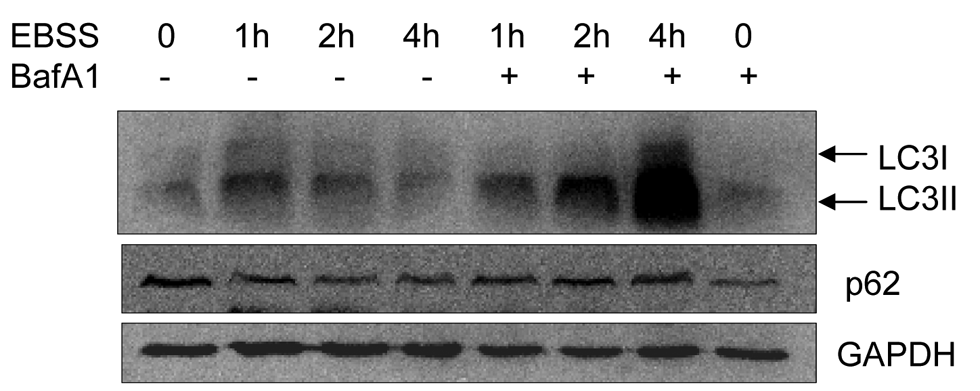

Supplement: Figure S1 — Flux analysis EBSS-induced autophagy in MEF. Wild-type MEF was cultured in EBSS for 1 h, 2 h and 4 h, with or without BafA1 (1 µM), followed by Western blotting for indicated proteins. Note the increase accumulation of LC33 in the presence of BafA1, which blocked the lysosomal degradation of the autophagosomes. This data showed the reduced level of LC3II in starved cells at later time points was due to increased autophagic degradation. (TIF) [file pone.0052347.s001.tif]

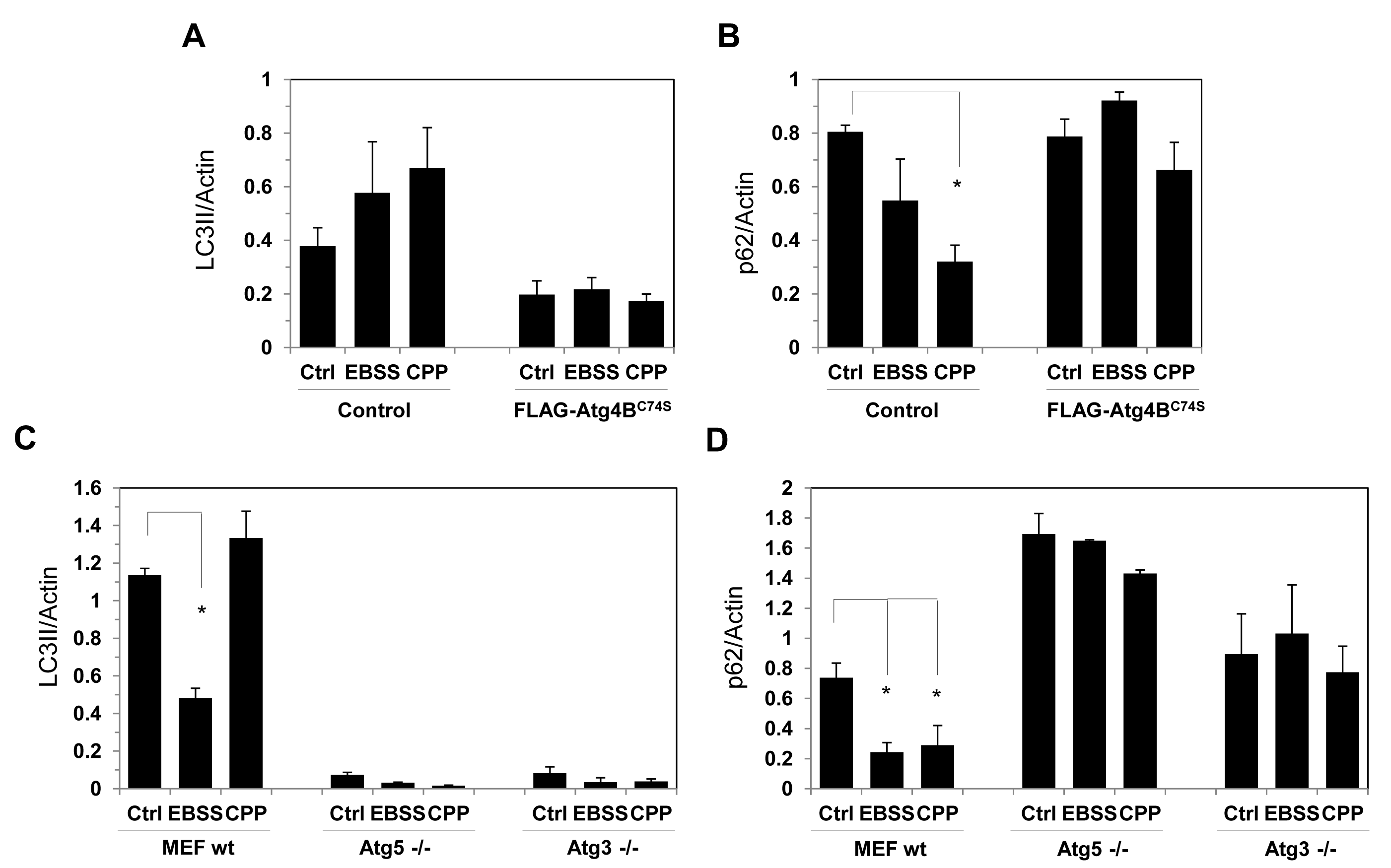

Supplement: Figure S2 — Quantitative measurements of LC3II and p62 in normal and autophagy deficient cells. (A–B) 293 cells expressing FLAG-Atg4BC74S or vector (control) were treated with CPP or EBSS. The levels of LC3II (A) or p62 (B) were determined by immunoblotting shown in Figure 1C, and were standardized to that of β-actin. (C–D) MEFs of different genotypes were treated with CPP or EBSS. The level of LC3II (C) or p62 (D) was determined by immunoblotting shown in Figure 1F and 1G, and were standardized to that of β-actin. Quantitative densitometry analysis was carried out from 2–4 independent experiments. Data were presented as mean±SEM. *: p<0.01. (TIF) [file pone.0052347.s002.tif]

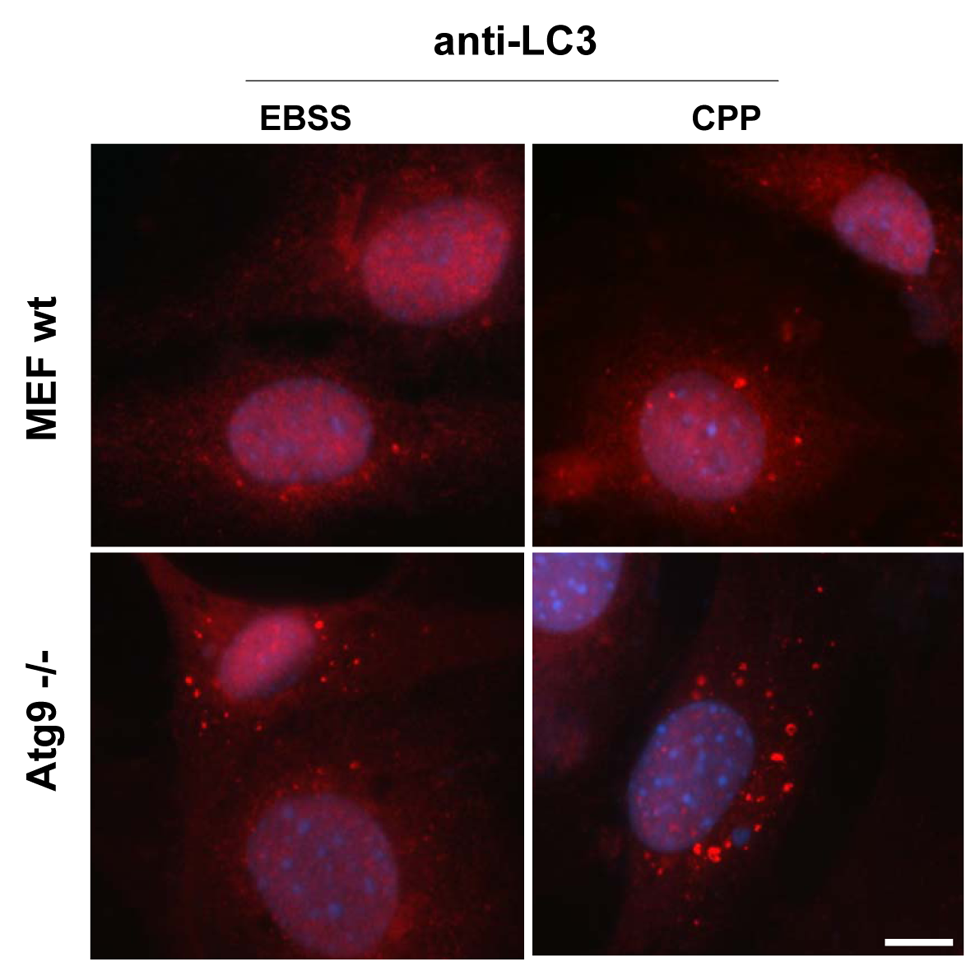

Supplement: Figure S3 — Atg9 is inhibitory for CPP-induced autophagy. Atg9 wt and Atg9−/− MEFs were treated with EBSS or CPP and then fixed. Formation of the endogenous LC3 puncta was detected by anti-LC3 immunostaining, counterstained with Hoechst 33342 for the nuclei. Scale bars: 10 µm. (TIF) [file pone.0052347.s003.tif]

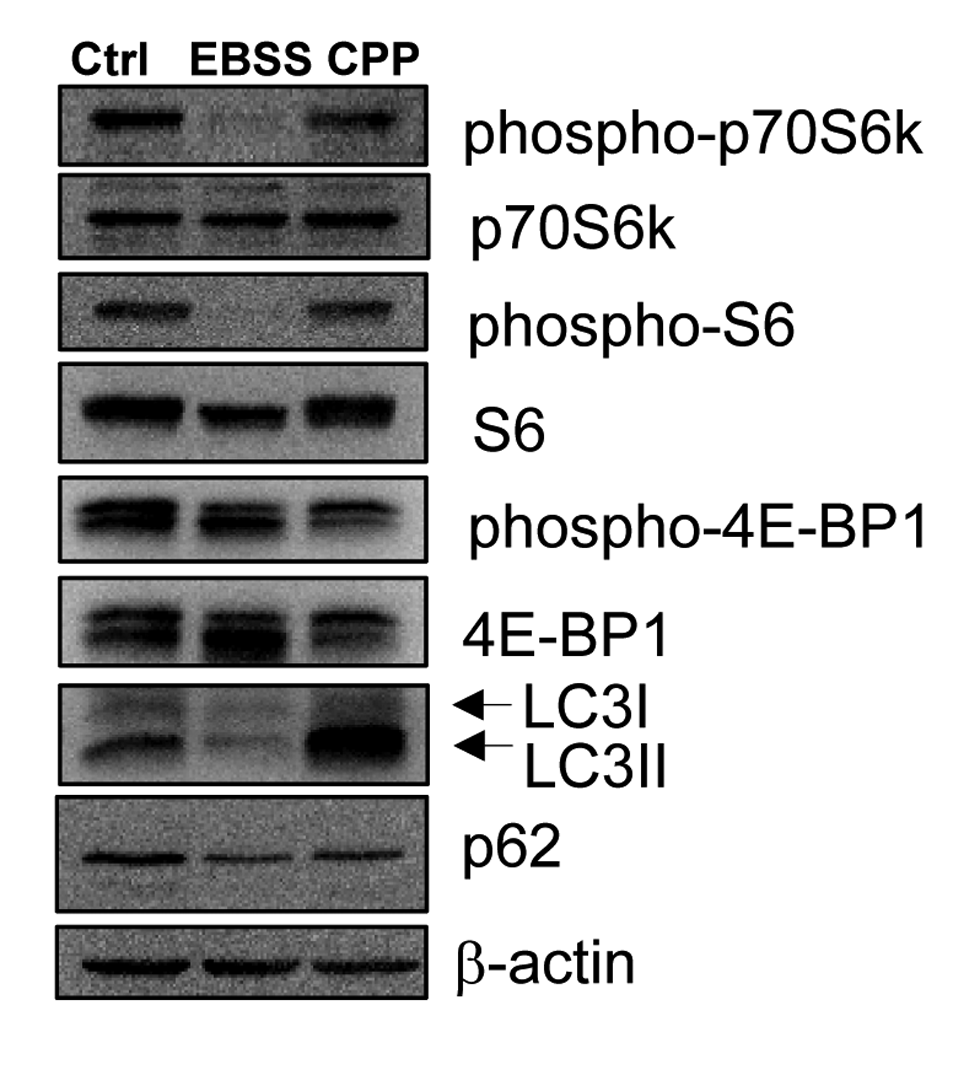

Supplement: Figure S4 — CPP-induced autophagy is independent of the signaling of mTOR. MEFs were treated with EBSS or CPP. Cells were solubilized on site in the presence of protease inhibitor and phosphatase inhibitors, followed by Western blotting with indicated antibodies. (TIF) [file pone.0052347.s004.tif]

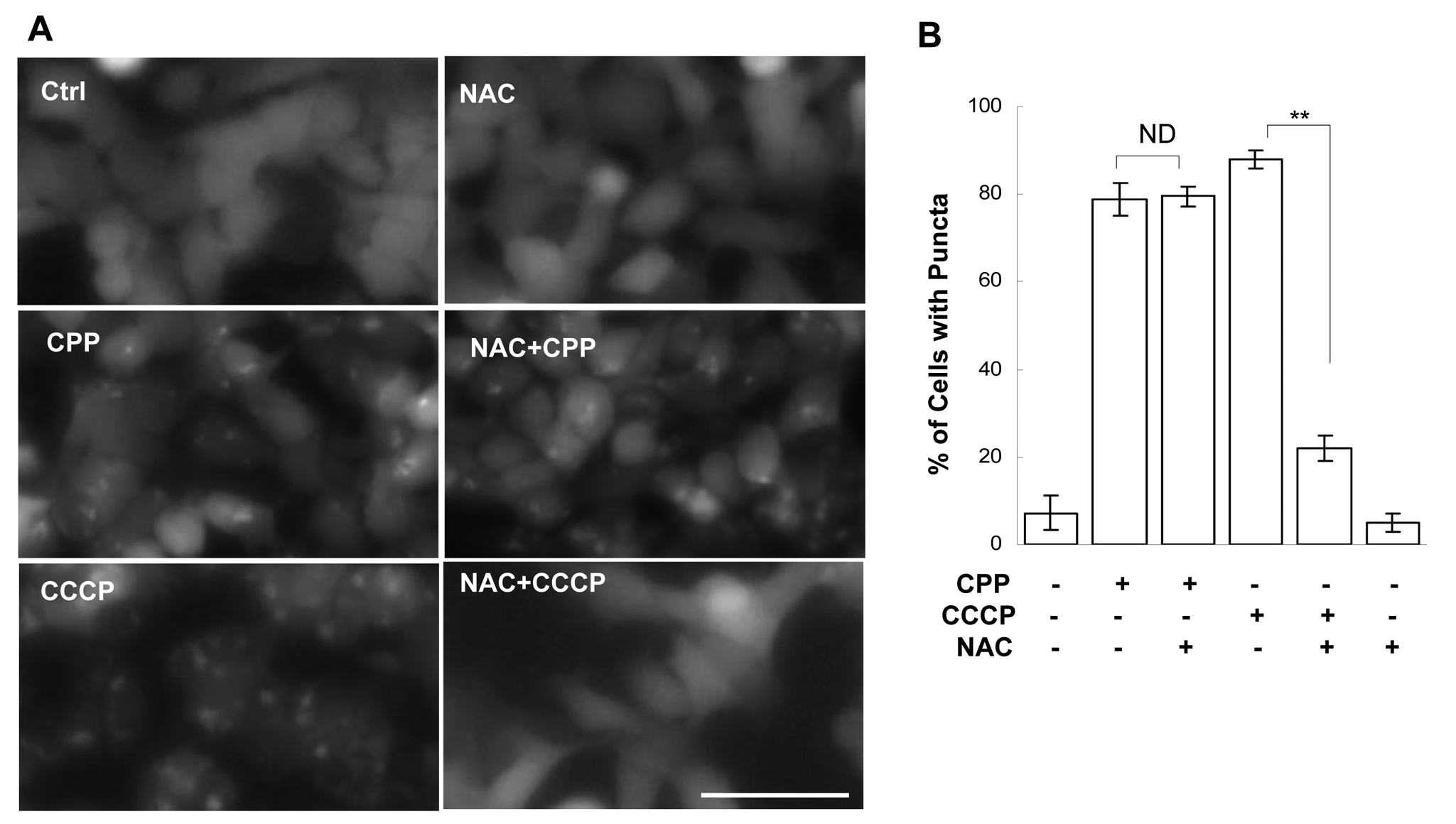

Supplement: Figure S5 — CPP-induced autophagy is independent of the signaling of ROS. (A) HEK293 cells stably expressing GFP-LC3 were treated with CPP or CCCP (40 µM) with or without NAC (10 mM) for 4 h. Images were acquired by fluorescence microscopy. Scale bars: 50 μm. (B) GFP-LC3 puncta was quantified from one representative experiment of three performed (mean ±SD). The data indicated that CCCP-induced, but not CPP-induced GFP-LC3 puncta formation could be blocked by NAC. Scale bars: 50 μm. *: p<0.01, ND: no statistical differences. (TIF) [file pone.0052347.s005.tif]

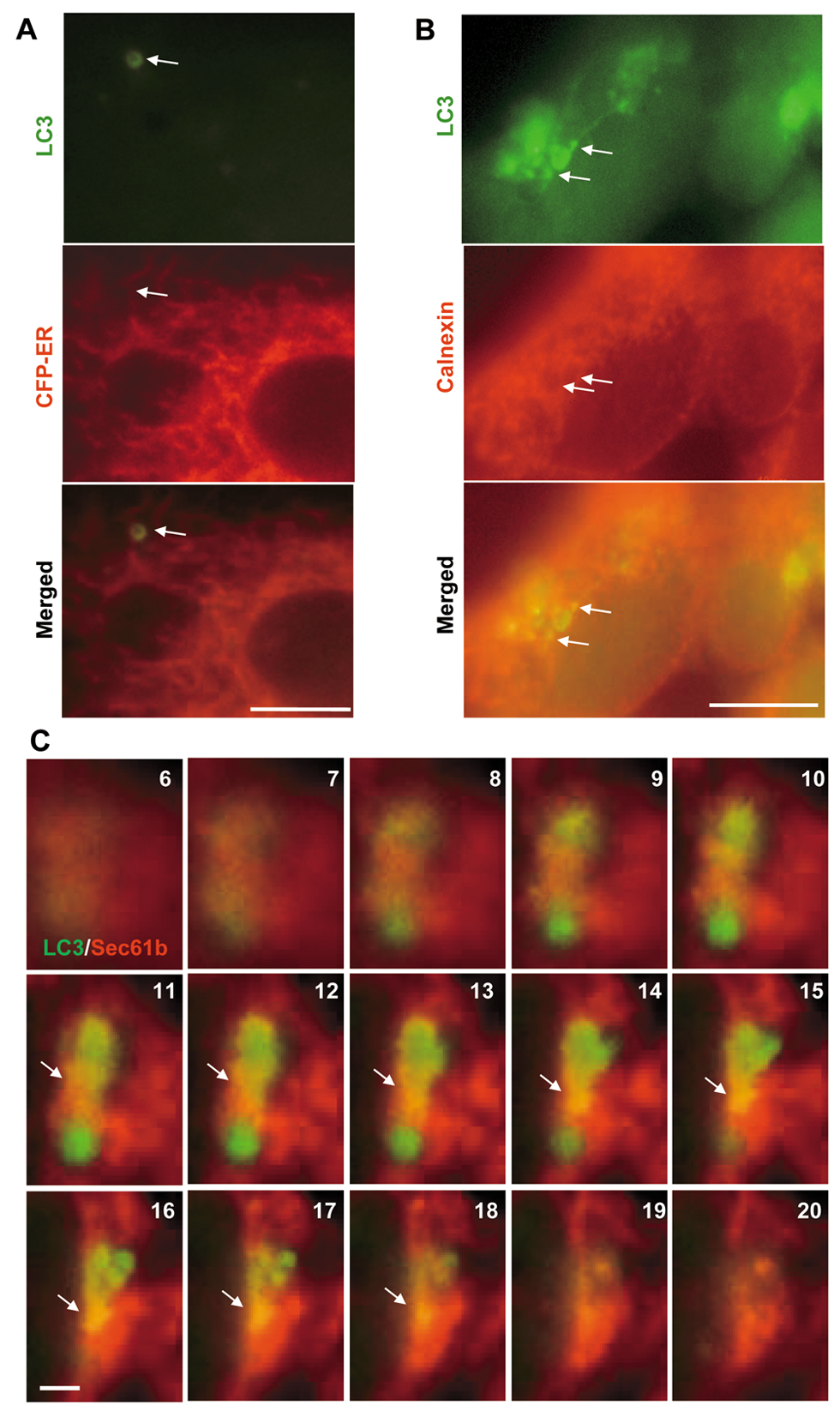

Supplement: Figure S6 — GFP-LC3 positive autophagosomes induced by CPP interact with ER membranes. (A) HEK293 cells stably expressing both GFP-LC3 and CFP-ER were treated with CPP. Fluorescent images were taken and CFP channel was pseudo-colored in red. (B) HEK293 cells stably expressing GFP-LC3 were treated with CPP, then fixed and stained with an anti-calnexin antibody and then Cy3-conjugated secondary antibody. (C) HEK293 cells stably expressing both GFP-LC3 and Sec61b-mCherry was treated with CPP. Images were taken at different z-sections (the section is indicated) and deconvoluted. Constructed z-stacks are shown in movie S2. Scale bars: 10 μm in A–B; 3 μm in C. Arrows indicate the colocalization of LC3 with CFP-ER, calnexin or Sec61b signals. (TIF) [file pone.0052347.s006.tif]
